# Low expression of FOXP2 predicts poor survival and targets caspase-1 to inhibit cell pyroptosis in colorectal cancer

**DOI:** 10.7150/jca.62433

**Published:** 2022-01-16

**Authors:** Peng Liao, Wu-hua Huang, Li Cao, Tao Wang, Li-ming Chen

**Affiliations:** Department of integration of traditional Chinese and Western Medicine and anorectum, the First Affiliated Hospital of Nanchang University, Nanchang, China

**Keywords:** colorectal cancer, FOXP2, caspase-1, cell pyroptosis

## Abstract

**Background:** Colorectal cancer (CRC) is the third most common cancer worldwide. Several studies have suggested that FOXP2 functions as a tumour suppressor. However, to date, it remains unclear how FOXP2 influences CRC occurrence.

**Methods:** We took advantage of CRC tissue samples and compared the expression of FOXP2 via immunohistochemistry assays. We elucidated the underlying function of FOXP2 in CRC cells by constructing a cell model with FOXP2 depletion. Co-IP experiments and immunofluorescence (IF) assays were conducted, and the results demonstrated that FOXP2 can promote the activation of caspase-1 to enhance cell pyroptosis.

**Results:** We used tissue RNA sequencing analysis in a colitis-associated cancer mouse model, and found that FOXP2 was downregulated in colitis and tumour tissues. We also found that CRC patients with low FOXP2 expression had poorer survival. Cell viability assays and electron microscopic examination showed that depletion of FOXP2 could enhance cell growth and inhibit cell pyroptosis. At the same time, knocking down FOXP2 expression was able to promote the protein expression of PCNA and cyclin D1 and downregulate the expression of the caspase family of proteins and GSDMD, which are markers of pyroptosis. A series of co-IP and IF assays revealed that FOXP2 interacts with caspase-1 and promotes its expression.

**Conclusion:** Our findings reveal a key role for FOXP2 in CRC cell pyroptosis and provide a mechanism explaining how FOXP2 promotes cell pyroptosis.

## Introduction

Colorectal cancer (CRC) is one of the most common tumours. According to the latest statistics in 2018, the incidence rate ranks fourth, and the mortality rate ranks third 1. At present, of all malignant tumours in China, the incidence and mortality of CRC ranks third and fifth, respectively, showing an obviously increasing trend 2. There are three pathways involved in the pathogenesis of sporadic CRC: the classical adenoma-adenocarcinoma pathway, the *de novo* pathway and the colitis-associated-carcinoma (CAC) pathway 1. It has been found that a series of genomic mutations or abnormal activation of molecular signalling pathways occur in the above processes, such as programmed cell death, which includes apoptosis, autophagy and pyroptosis 3. During the progression from colitis to CRC, the function of cell pyroptosis is complex; on the one hand, pyroptosis is able to inhibit the occurrence and development of tumours; on the other hand, as a type of proinflammatory death, pyroptosis can form a suitable microenvironment for tumour cell growth and thus promote tumour growth 4. Moreover, great advances in understanding the pathogenesis of CRC have been achieved over the past several decades; however, the prognosis of CRC patients remains poor due to high malignancy. Consequently, a better understanding of the molecular mechanisms underlying CRC would promote the development of effective therapeutic strategies for CRC patients.

Forkhead box family proteins (FOXPs) are a group of highly conserved proteins comprised of a C2H2 zinc finger domain, leucine zipper domain (WHD), wing helix forkhead DNA binding domain (FHD) and an approximately 50-residue N-terminal domain. FOXP family proteins consist of four members, namely, FOXP1, FOXP2, FOXP3 and FOXP4 5. The structure of the FHDs of FOXP family proteins includes a C-terminal helix forkhead structure, but they are different across the different members 6. The dimer structure of FOXP3 is more stable. FOXP family proteins were initially found in T cells, and they can regulate the role of T cells in the development of the thymus 6. For example, FOXP3 overexpression in Treg cells can inhibit the tumour immune response and promote the proliferation of tumour cells 7. Similarly, FOXP1 can be coupled with FOXP3 and inhibit DNA transcription activity. More studies have found that FOXP1 and FOXP2 are expressed in lung tissue, and FOXP1 plays an antitumour role in the development of lung cancer 8. FOXP2 is considered to play an antitumour role in a variety of lymphomas, such as multiple myeloma, gastric cancer and liver cancer 9. However, the function of FOXP2 in CRC is unknown.

Here, in our study, through performing RNA-sequence analysis of colitis-associated CRC constructed by AOM and DSS reagents, we found that FOXP2 was a potential target for preventing carcinogenesis. Using *in vitro* assays, we elucidated that low expression of FOXP2 in CRC prevents pyroptosis by downregulating caspase-1 expression. Additionally, we found that lower FOXP2 expression predicted poorer prognosis of CRC patients.

## Materials and Methods

### Patients and tissue sample

In our study, there were two types of colorectal mucosal tissues, namely, those obtained by surgery and those obtained by endoscopy. In total, 83 pairs of surgical tissues were fixed by formalin and embedded in paraffin (COC1601, Shanghai Superbiotech Pharmaceutical Technology, Shanghai, China) and then used to perform tissue microarray and immunohistochemistry (IHC) analyses. All patients provided signed informed consent and had not undergone preoperative treatment. The inclusion criteria were as follows: (1) patients who were diagnosed with CRC by pathology and who were over 20 years old; and (2) patients who did not receive preoperative adjuvant therapy. The exclusion criteria included patients with severe diseases such as cirrhosis, renal failure, and cardiac failure. Fifteen pairs of endoscopic mucosal tissues obtained from the First Affiliated Hospital of Nanchang University were used to perform real-time quantitative PCR analyses to measure the levels of FOXP2 and caspase-1 mRNA. All patients signed the informed consent form. Detailed information on the patients who underwent surgery is shown in Table 1.

### Bioinformatics analysis

Total RNA was isolated from each thymic sample using the standard TRIzol protocol (Invitrogen, Carlsbad, CA, USA). The RNA quality was examined by gel electrophoresis and with a NanoDrop spectrophotometer (Thermo, Waltham, MA, USA). For RNA sequencing, RNA samples from 6 biological replicates were separated into two independent pools, each comprised of three distinct samples of equal amounts. Strand-specific libraries were constructed using the TruSeq RNA sample preparation kit (Illumina, San Diego, CA, USA), and sequencing was carried out using the Illumina HiSeq X Ten instrument by the commercial service organization Energy Biotechnology Co., Ltd. (Shanghai, China). As described in a previous study 10, 11, the expression of the transcripts was calculated by the FPKM method using Perl. Differentially expressed transcripts (DETs) were determined using the MA-plot-based method with Random Sampling (MARS) model in the DEGseq package between different time points. Then, DETs were chosen for functional and signalling pathway enrichment analysis using the GO and KEGG databases. In addition, GSE44904 and GSE44988 were used to analyse the differentially expressed genes via R software. The significantly enriched pathways were determined when P<0.05 and at least two affiliated genes were included.

### Cell culture and transfection

Our study was conducted using the HCT116 and SW480 cell lines, which were purchased from American Type Culture Collection. The cell lines were cultured as previously described 12. Briefly, CRC cells were cultured in RPMI-1640 medium (purchased from Gibco Company, USA) mixed with 10% FBS, 100 U/ml penicillin and 50 mg/ml streptomycin. The cells were incubated at 37°C in a humidified atmosphere containing 5% CO2. Cell transfection was completed by Lipofectamine™ 2000 (Invitrogen, Carlsbad, CA, USA), and cotransfection was achieved by FuGENE HD (Promega) according to the manufacturer's instructions. The siRNA sequences were as follows: sense sequence, 5'-3' GCGACAGAGACAAUAGCATT, anti-sense sequence, 5'-3' UGCUUAUUGUCUCUGUCGCTT; sense sequence, 5'-3' GGACAGUCUUCAGUUCUAATT, anti-sense sequence, 5'-3' UUAGAACUGAAGACUGUCCTT.

### Cell proliferation (MTT)

Ninety-six-well plates and 6-well plates were used to perform the MTT assay and colony formation assay, respectively. For the MTT assay, CRC cells were incubated in plates at a density of 10^3 cells. Then, the cells were transfected with FOXP2 siRNA. Cell viability was measured at 490 nm after incubation with MTT reagents for 4 hours at 37°C.

### Western Blot

All proteins were extracted by RIPA buffer (Solarbio Life Science, Beijing, China) mixed with protease inhibitors on ice plates. Then, protein concentration was qualified by a BCA protein assay kit purchased from Solarbio Life Science. Next, equal amounts of protein were loaded onto 10% SDS-PAGE gels and transferred to nitrocellulose membranes (Millipore). The membranes were blocked with 5% BSA (Sigma-Aldrich) and incubated with primary antibodies at 4°C overnight. On the second day, the membranes were washed with 1x TBS solution and incubated with the secondary antibody conjugated with HRP at normal temperature for 1 hour. Finally, the results were determined using the ChemiDoc^TM^ Imaging System (Bio-Rad, Hercules, CA, USA). The following primary antibodies were used: FOXP2 (20529-1-AP, 1:1000, ProteinTech), PCNA (ab29, 1:1000, Abcam), cyclin D1 (ab40754, 1:5000, Abcam), caspase-1 (sc-392736, 1:1000, Santa Cruz), caspase-3 (sc-7272, 1:1000, Santa Cruz), caspase-9 (ab32539, 1:1000, Abcam), GSDMD (ab209845, 1:1000, Abcam), GAPDH (1:1000, TransGen Biotech, Beijing), Flag-tag (D6W5B, CST), HA-tag (C29F4, CST), and β-actin (1:1000, TransGen Biotech, Beijing). The secondary protein was purchased from TransGen Biotech (Beijing).

### Co-immunoprecipitation

A co-immunoprecipitation (Co-IP) experiment was performed to determine if the FOXP2 and caspase-1 proteins interact. CRC cells were seeded in 10 cm plates and then scraped with RIPA lysis buffer. A volume of 1 μL primary antibody, 50 μL Protein A-Agarose (sc-2003; Santa Cruz Biotechnology, Dallas, TX, USA), and 500 μL phosphate-buffered saline (PBS) was added and the cells were incubated for 2 h. Next, the cells were incubated with primary antibody overnight at 4°C on a rocking platform. The primary antibodies targeted FOXP2, caspase-1, and IgG (BL003A, Bio-Sharp, Shanghai, China). The next day, the cells were centrifuged at 3,000 rpm and 4°C, washed twice with PBS containing protein inhibitors, followed by Western blotting with the same primary antibodies used for the Co-IP experiments.

### Immunohistochemistry (IHC)

CRC tissue was warmed at 70°C for two hours, dewaxed with xylene and anhydrous ethanol for 40 min, and incubated in citrate to complete antigen retrieval. Finally, tissue microarrays were incubated with primary antibodies overnight at 4°C. On the second day, the microarrays were incubated with the corresponding secondary antibody for 30 min at room temperature. After washing with PBS, the tissue microarrays were stained with DAB reagent (TransGen Biotech, Beijing), and the nuclei were stained with haematoxylin. The IHC results were assessed by a method described previously 12, 13. FOXP2 and caspase-1 staining was semiquantitatively assessed using a grade scoring system according to the intensity of staining (scored as 0, no staining; 1, weak staining; 2, moderate staining; 3, strong staining) and the percentage of positive tumour cells (scored as 0, none; 1, 1%-29%; 2, 30%-69%; 3, >70%). The total IHC score of each tissue sample was calculated to determine the cut-off value for the low and high expression groups by multiplying the staining intensity score by the positive tumour cell score. The final score, which ranged from 0-9, was defined as follows: 0, negative; 1-3, weak; 4-6, moderate; and >6, strong. Therefore, FOXP2 expression was sorted into 2 categories: high level (grades 4-9) and low level (grades 0-3). All staining results were scored by two independent pathologists in a blinded manner.

### Cell immunofluorescence assay

CRC cells were incubated in chamber slides for 24 h, fixed in 4% paraformaldehyde for 20 min, and permeabilized with 0.1% Triton X-100 for 1 h. Next, the cells were incubated with FOXP2 (ab16046, 1:100 Abcam) and caspase-1 (sc-392736, 1:100, Santa Cruz) primary antibodies overnight at 4°C. Finally, the cells were incubated with goat anti-rabbit or anti-mouse IgG fluorescent secondary antibody (Thermo Fisher Scientific), and the nuclei were stained with DAPI. The intensity of immunofluorescence was examined by confocal fluorescence microscopy at wavelengths of 488 and 594 nm.

### Real-time quantitative PCR

The tissue was lysed by TRIzol reagent (TransGen Biotech, Beijing) and total RNA was extracted by chloroform and isopropyl alcohol. Then, the concentration of RNA was measured by a NanoDrop 2000 (Thermo Scientific, Wilmington, DE, USA). cDNA synthesis and quantitative PCR was completed according to the instructions of the reagents purchased from TransGen Biotech. The FOXP2 primer sequences were as follows: forward 5'-3' AGCTCTGAAGTAAGCACAGTAG and reverse 5'-3' TGCTGCTGTAAAAGAAGTTGTC. The GAPDH primer sequences were the same as those previously reported 12.

### Electron microscopic examination

To examine the morphology of pyroptotic cells, cells were first seeded in 35-mm culture dishes. After transfecting FOXP2 siRNA or plasmid for 48 hours, we collected cells in PBS solution and then fixed the cells with glutaraldehyde purchased from Solarbio Life Sciences (China). Static bright-field images were captured using a Leica XSP-8CA microscope. The pore-forming activity in lobaplatin-induced pyroptosis was examined by transmission electron microscopy (TEM).

### Chromatin immunoprecipitation

Chromatin immunoprecipitation (ChIP) assays were performed according to the instructions of the ChIP assay kit (17-295, Millipore, USA). Briefly, we seeded 1X10^^6^ cells on 10-cm dishes, fixed them with 1% formaldehyde (diluted from a 37% formaldehyde solution), and added 1 ml of a 1X glycine solution. Next, the cell pellet was lysed with SDS lysis buffer and sonicated into 200-500 base pair fragments. Protein Agarose Beads (50 μL) and anti-FOXP2 antibody (4 μg) were added to the sonicated cell supernatant to immunoprecipitate the chromatin. Finally, the immunoprecipitated DNA was amplified as described in the literature 11. The sequence for the caspase-1 promoter is listed in supplementary material 1. FOXP2 (ab16046, Abcam) was used for the ChIP assay, and rabbit IgG was used as a control group.

### Luciferase reporter assay

As in our previous study 11, 1,500 cells were seeded into a plate for the luciferase assay. The Luc-Pair^TM^ Duo-Luciferase Assay Kit 2.0 was purchased from GeneCopoeia (China). A caspase-1 promoter plasmid (1 μg) or a truncated promoter plasmid was cotransfected into the cells with 1 μg firefly plasmid. After 24 h, luciferase activity was examined using FlUOstar Omega. The vector pGL3 was used in the control group.

### Statistical analysis

As previous studies described, the statistical analyses were performed using SPSS and GraphPad Prism 8 software. The chi-squared test was used to investigate the relationship between FOXP2 expression and clinicopathological factors. Univariate and multivariate Cox regression analyses were used to identify risk factors for prognosis. Survival curves of FOXP2 and caspase-1 expression were plotted by the Kaplan-Meier method and compared using the log-rank test. Two-tailed Student's t-tests were used to assess the differences between the control group and treatment group. All assays were repeated more than twice. The differences were considered to be statistically significant when *P*<0.05.

## Results

### Low expression of FOXP2 predicted poorer prognosis in CRC

**Figure 1:**
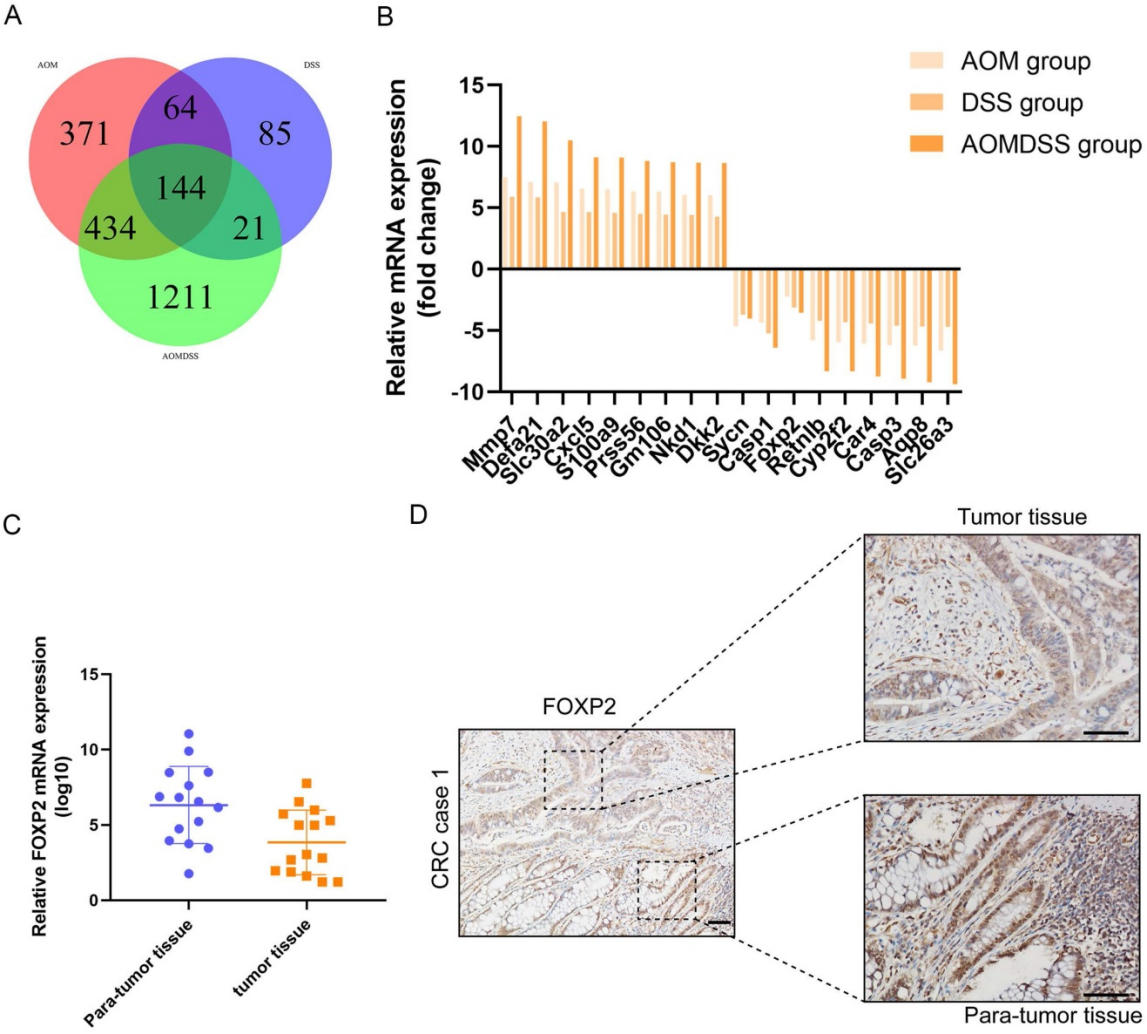
** FOXP2 was downregulated in the colitis-associated cancer model and colorectal cancer. A** Venn plot shows the coexpression of different genes among the three groups. **B** Top ten genes according to the fold change. **C** RT-PCR assay was performed to measure the level of FOXP2 mRNA in CRC tissues. **D** IHC was performed to measure the level of FOXP2 protein in CRC tissues.

**Figure 2:**
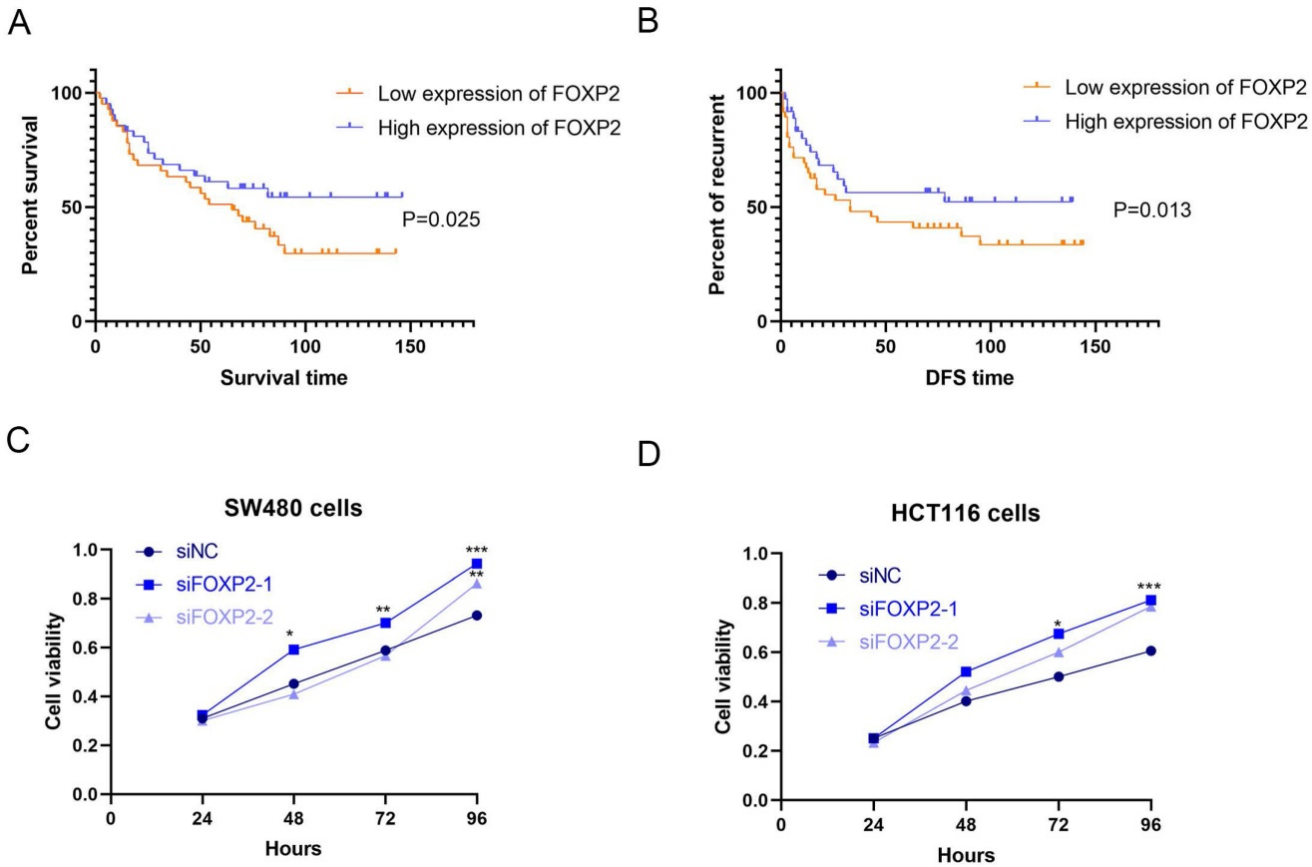
** Low expression of FOXP2 was associated with poor prognosis of patients, inhibited cell growth and promoted cell pyroptosis.** K-M survival curves (**A**) and disease-free survival curves (**B**) were generated to assess the effect of FOXP2 expression in CRC patients. **C-D** MTT assay was performed to explore the viability of CRC cells after depleting FOXP2. **E** Electron microscopic examination was performed to assess cell pyroptosis after depleting FOXP2.

In our study, to identify the differentially expressed genes, we performed transcriptome analysis using an AOM/DSS mouse model supported by a GEO dataset (GSE44904 and GSE44988). As the results show, we identified the differentially expressed genes in the AOM alone-, DSS- or AOM/DSS-treated groups (Supplementary Figure 1A-1C). Analysis of the GEO dataset revealed that 144 genes were differentially expressed among the three groups (Figure 1A), among which FOXP2 was downregulated in the treatment group (Figure 1B). Additionally, we found that caspase family members, such as caspase-1 and caspase-3, were downregulated in all three groups (Figure 1B). To further demonstrate our findings, we collected 15 pairs of CRC paratumour and tumour tissues by endoscopy, and found that FOXP2 was downregulated in the tumour tissues compared to the paratumour tissues according to RT-PCR assay (Figure 1C). Moreover, surgical samples were collected for IHC assays and the results similarly showed that FOXP2 protein was overexpressed in tumour tissues (Figure 1D). In line with our RT-PCR assay results, we found lower expression of FOXP2 in the tumour tissue. To analyse the effect of overexpression of FOXP2 on prognosis, survival information was compared with the IHC assay result obtained from a tissue microarray of 83 pairs of CRC tissues. According to the IHC assay results, we divided all patients into two groups, namely, the FOXP2 overexpression group and the FOXP2 low-expression group (Table 1). The chi-square test showed that FOXP2 expression was associated with TNM stage and tumour size (P<0.05). Univariate and multivariate Cox analyses found that FOXP2 was an independent risk factor for prognosis, as well as T stage, TNM stage and distant metastasis (Table 2). The K-M survival curve showed that lower expression of FOXP2 predicted poorer survival (Figure 2A). The disease-free survival curve also showed that patients with lower expression of FOXP2 had a higher recurrence rate (Figure 2B).

### FOXP2 inhibits cell growth and promotes cell pyroptosis in colorectal cell lines

**Figure 3:**
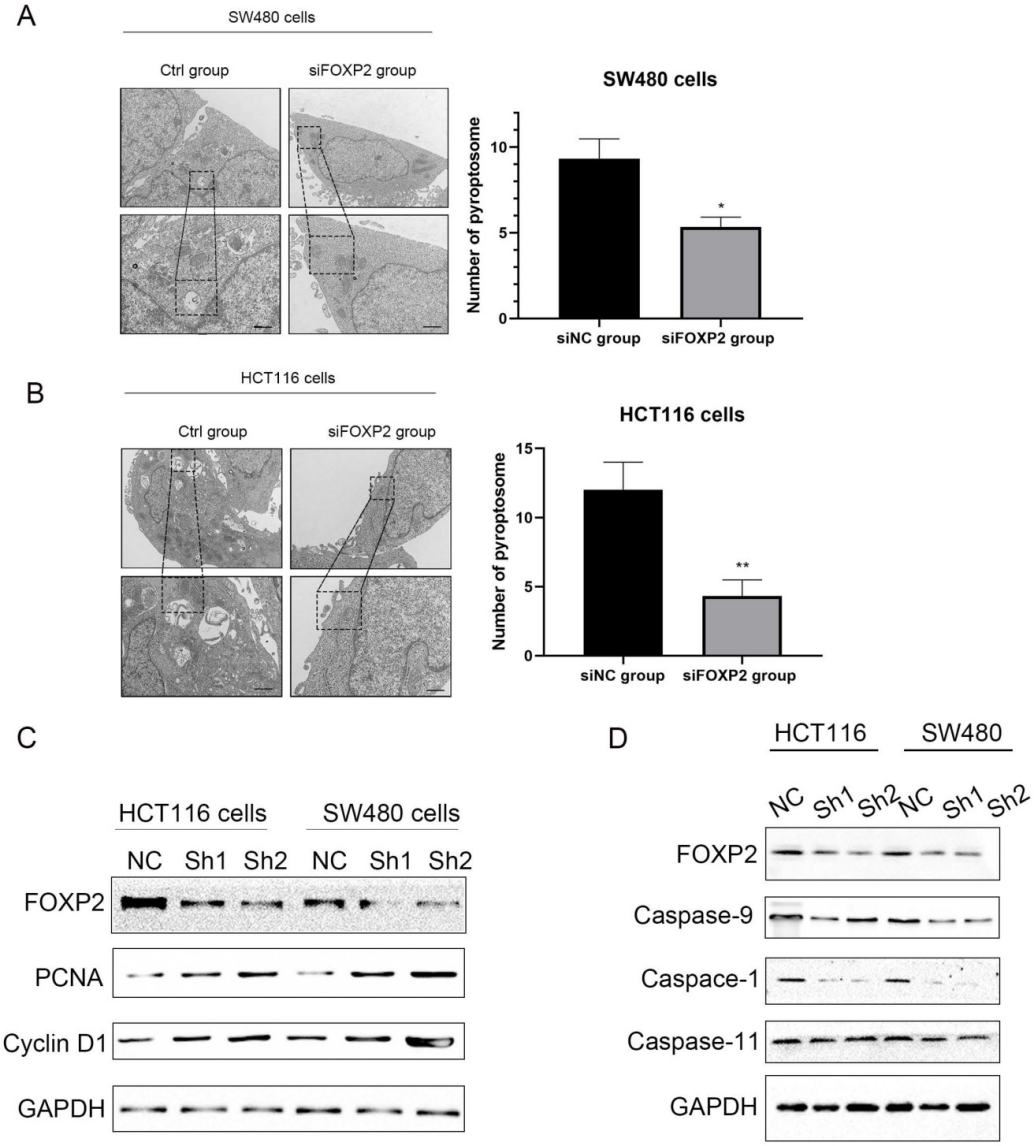
** Depletion of FOXP2 expression was correlated with cell pyroptosis.** Electron microscopic examination was performed to assess cell pyroptosis after depleting FOXP2.** B** Western blotting was performed to measure several proteins associated with cell proliferation, such as PCNA and Cyclin D1, when FOXP2 was depleted in HCT116 and SW480 cells. **C** Some proteins, such as caspase-9, caspase-1 and caspase-11, which are related to cell pyroptosis, were measured following FOXP2 knock down.

Based on the previous results, we thought that the FOXP2 gene could inhibit carcinogenesis. Therefore, we performed a functional assay to test our hypothesis. As shown by the MTT assay, we constructed a FOXP2 depleted cell model and measured cell viability, and the results suggested that cell viability in both SW480 cells and HCT116 cells was enhanced after knocking down FOXP2 expression (Figure 2C-2D). Considering that caspase-1 was downregulated in the colitis-associated cancer mouse model and caspase-1 plays a central role in cell pyroptosis, we explored cell death by TEM. As shown in Figure 3A-3B, compared to the FOXP2 depletion group, cells in the control group more frequently exhibited hole formation, cell swelling and cytoplasmic outflow, suggesting that pyroptosis more easily occurred in cells with high FOXP2 expression. Furthermore, we measured some proteins associated with cell growth and cell pyroptosis via Western blot assay. The results revealed that proteins associated with cell proliferation, such as PCNA and cyclin D1, were increased when FOXP2 expression was knocked down in both HCT116 cells and SW480 cells (Figure 3C). Similarly, we measured the proteins correlated with pyroptosis and found that caspase-1, caspase-9 and caspase-11 were decreased as FOXP2 was depleted (Figure 3D). These results suggested that the expression of FOXP2 could prevent cell proliferation and cell pyroptosis.

### Caspase-1 is the downstream factor of FOXP2 that regulates cell pyroptosis

**Figure 4:**
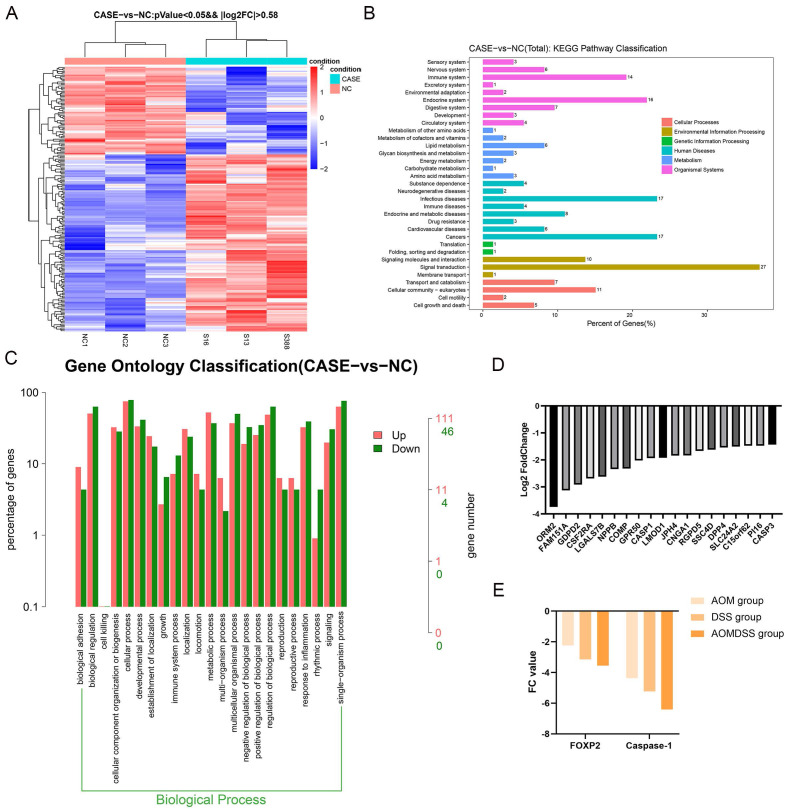
** Depletion of FOXP2 was correlated with the expression of cell pyroptosis genes. A** Heat plot was generated to show differentially expressed genes by RNA sequencing (case group indicates the FOXP2 knock down group, while NC group indicates the control group)**. B-C** KEGG pathway and GO analyses were performed according to the differentially expressed genes as determined by RNA sequencing. **D** Top ten genes according to the fold change. **E** The relationship between FOXP2 and caspase-1 among the AOM, DSS and AOM/DSS groups.

**Figure 5:**
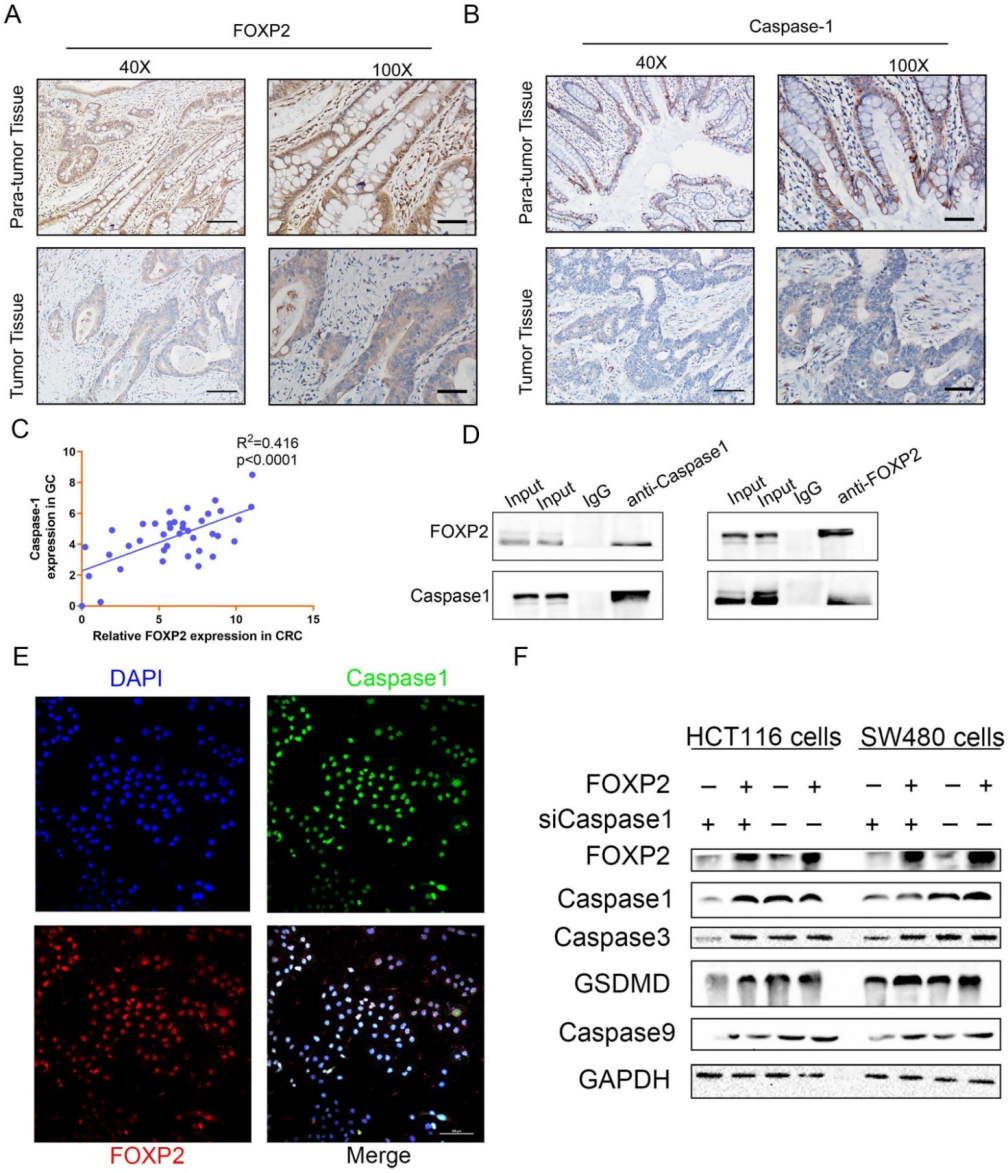
** FOXP2 promoted cell pyroptosis by interacting and regulating caspase-1. A-B** The association of FOXP2 with caspase-1 in CRC tissues was assessed using IHC assays. **C** The relationship between FOXP2 and caspase-1 mRNA levels was measured via RT-PCR**. D** The interaction between endogenous FOXP2 and caspase-1 was detected by co-immunoprecipitation assays with anti-FOXP2 and anti-caspase-1 antibodies in HCT116 and SW480 cells. Immunoglobulin G (IgG) antibody was used as the control, and cell lysates were used to examine FOXP2 and caspase-1 expression. **E** Immunofluorescence assays were performed to assess colocalization between FOXP2 and caspase-1 in SW480 cells. **F** Western blotting was performed to measure proteins related to cell pyroptosis, such as caspase-3, caspase-1, GSDMD and caspase-9, in HCT116 and SW480 cells cotransfected with FOXP2 plasmid and caspase-1 siRNA.

**Figure 6:**
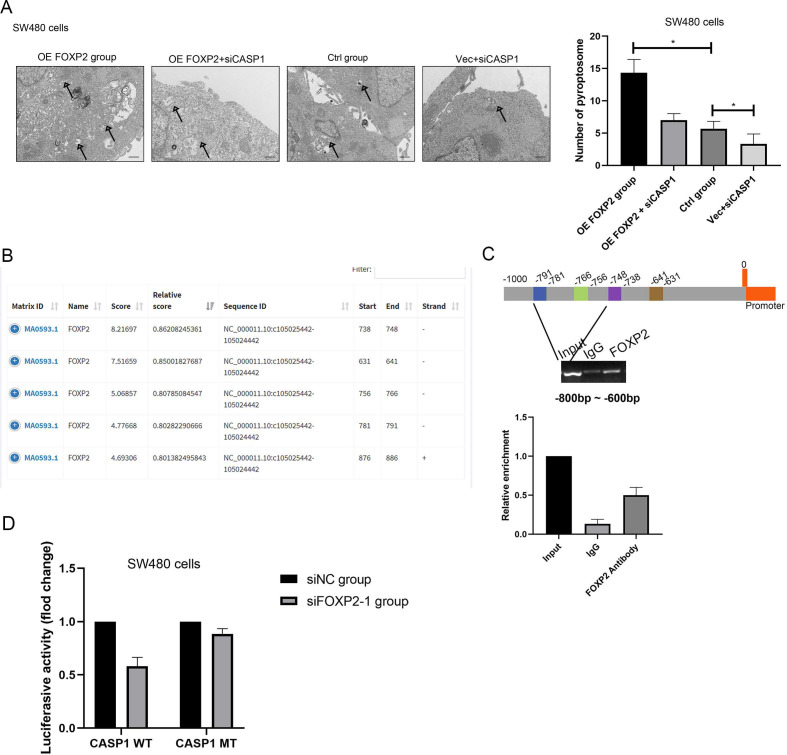
** FOXP2 promoted cell pyroptosis by promoting the transcription of caspase-1. A** Alterations in cell pyroptosis were compared via electron microscopic examination after overexpression of FOXP2 or depletion of caspase-1. Representative pictures are on the left, while the results are shown on the right. **B** Three potential FOXP2 binding sites in the promoter region of caspase-1 according to the JASPAR database (http://jaspar.genereg.net/). **C** ChIP assays were performed in SW480 cells to investigate whether FOXP2 could bind the promoter of caspase-1, followed by qPCR amplification of the suggested binding site within the caspase-1 promoter region.** D** A luciferase reporter assay was conducted to determine that FOXP2 could regulate caspase-1 transcription. *P<0.05, ***P<0.001. All data are presented as the mean ± SD, and the experiments were repeated three times. Data represent the average of 3 independent experiments.

To investigate the downstream factors of FOXP2, we performed RNA sequencing using SW480 cells with FOXP2 depletion. Briefly, we collected 3 groups transfected with control siRNA and 3 groups transfected with FOXP2 siRNA and performed transcriptome analyses. The differentially expressed genes were shown in a heat map and KEGG pathway classification was performed (Figure 4A-4C). We found that the differentially expressed genes were involved in the process of pyroptosis (Figure 4D). According to the fold change value, we found that some genes were positively associated with FOXP2 expression, among which we found that caspase family members, including caspase-1 and caspase-3, were downregulated (Figure 4D). In line with this result, caspase-1 was also found to be positively related to FOXP2 expression in a mouse model (Figure 4E). To demonstrate this relationship, we performed an IHC assay and found that the FOXP2 protein was positively associated with the caspase-1 protein (Figure 5A-5B, Table 1). Additionally, FOXP2 mRNA expression was positively correlated with caspase-1 mRNA expression, which was confirmed by RT-PCR assay (Figure 5C). In addition, we performed a CO-IP assay and found that FOXP2 interacted with caspase-1 (Figure 5D), which was consistent with the immunofluorescence assay results (Figure 5E). For the rescue assay, we found that depletion of caspase-1 was able to impair the effect of FOXP2 overexpression on cell pyroptosis, which was confirmed by the result of proteins such as caspase-3, caspase-9 and GSDMD, cell pyroptosis status markers (Figure 5F). Hence, our results showed that low expression of FOXP2 could prevent cell pyroptosis by decreasing the expression of caspase-1. Since FOXP2 is a transcription factor, we hypothesized that FOXP2 mediates caspase-1 expression by binding to its promoter. To explore the mechanism of transcriptional regulation between FOXP2 and caspase-1, we applied the online software program JASPAR to predict the potential FOXP2 binding sites in the caspase-1 promoter. As shown in Figure 6B, four possible binding sites were found. Next, we determined the -1000 bp to +1 bp sequence via the online software program NCBI and designed a 200 bp primer (supplementary material 1). According to the ChIP assay results (Figure 6C), FOXP2 could bind the region between -800 bp and -600 bp. Based on these results, we constructed a wild-type plasmid and mutant plasmid for the caspase-1 promoter region and measured luciferase activity after transfecting these plasmids into SW480 cells. The results showed that overexpression of FOXP2 enhanced the activity of the caspase-1 WT plasmid, but this effect was not observed when the caspase-1 mutant plasmid was transfected (Figure 6D). Taken together, these results indicate that FOXP2 directly binds the caspase-1 promoter region and ultimately promotes cell pyroptosis.

## Discussion

Pyroptosis is an inflammatory form of cell death triggered by certain inflammasomes, leading to the cleavage of gasdermin D (GSDMD) and activation of inactive cytokines such as IL-18 and IL-1β^14^. Pyroptosis has been reported to be closely associated with carcinogenesis, especially for tumours induced by inflammation. Recently, some studies found that pyroptosis can influence cell proliferation, invasion and metastasis of tumours, which are regulated by some noncoding RNAs and other molecules 4. However, the correlation between pyroptosis and cancer is complex. On the one hand, pyroptosis can inhibit the occurrence and development of tumours. On the other hand, as a type of proinflammatory death, pyroptosis can form a suitable microenvironment for tumour cell growth and thus promote tumour growth 4. Hence, we provided a new mechanism for pyroptosis regulation. In our study, to explore potential markers contributing to the prevention and treatment of CRC, we found that FOXP2 promotes caspase-1 expression to inhibit the proliferation of CRC cells and that FOXP2 plays a potential role in CRC.

As transcription factors, the FOXP family members include FOXP1, FOXP2, FOXP3 and FOXP4, among which FOXP2 was first identified as an important gene controlling speech formation^6^. Accumulating evidence shows that FOXP proteins have important functions in the regulation of carcinogenesis and the immune response. For instance, FOXP1 enhances breast cancer cell motility by interacting with NFAT1, and FOXP2 activates p21 expression to promote growth arrest of osteosarcoma cells 15, 16. In line with previous results 9, 17, in our study, we found that FOXP2 functions as a tumour suppressor and can be used to predict patient prognosis. Further investigation of the mechanism via RNA-sequencing analysis found that FOXP2 is associated with cell pyroptosis. In detail, FOXP2 promotes the expression of caspase-1, which plays a crucial role in regulating cell pyroptosis, and FOXP2 interacts with caspase-1. Consistent with our results, other studies have also reported that FOXP proteins are correlated with caspase-1^18^-^20^. FOXP1 has been shown to function as a gatekeeper of vessel inflammation by directly regulating endothelial inflammasome components, including caspase-1^18^. Additionally, most studies consider FOXP2 to be a tumour suppressor; however, in several lymphomas, FOXP2 functions as an oncogene upregulates the levels of angiogenic factors such as VEGF with G patch and FHA domains 6, 21. The dual function of FOXP2 depends on the related signalling pathways. Furthermore, FOXP2 can act as a transcription factor in brain development and function, as well as in lymphatic vessel development 22, 23. Many downstream factors are regulated by FOXP2, such as Lnc PRAT1 and TGF-β 24. In our study, we found that FOXP2 could bind the region between -800 bp and -600 bp of the caspase-1 promoter, thereby regulating caspase-1 mRNA transcription.

In conclusion, our results suggest that FOXP2 expression is downregulated in CRC tissues and that reduced FOXP2 expression is associated with poor overall survival. In addition, downregulation of FOXP2 significantly reduced cell pyroptosis, which was due to inhibition of caspase-1 expression. These findings reveal that FOXP2 might be a new prognostic factor and be closely correlated with CRC cell pyroptosis.

## Supplementary Material

Supplementary figure and information.

## Figures and Tables

**Table 1:** Correlation of FOXP2 expression with the clinicopathological characteristics of 83 CRC patients

FOXP2 expression				
Characteristic	Category	Low (n=49.4%)	High (n=50.6%)	*P* value
Age	≤50	11	12	0.859
	>50	30	30	
Gender	Male	25	24	0.821
	Femal	16	18	
TNM staging	I/II	11	21	*0.03*
	III/IV	30	21	
Pathological grade	I	7	1	*0.021*
	II	30	40	
	III	4	1	
Lymph node metastasis	No	24	27	0.591
	Yes	17	15	
Tumor size	≥5cm	20	30	*0.035*
	<5cm	21	12	
T stage	T1/T2	9	6	0.364
	T3/T4	32	36	
Distant metastasis	No	20	25	0.326
	Yes	21	17	
CEA	Normal	14	15	0.881
	High	27	27	
CA199	Normal	3	6	0.307
	High	38	36	
Caspase-1 expression	Low	26	15	*0.011*
	High	15	27	

**Table 2:** Univariate and multivariate cox regression of prognostic factors for survival of patients with CRC.

	Univariate analysis		Multivariate analysis	
Characteristic	HR value	P value	HR value	P value
Age				
<=50	Reference	-		
>50	0.816(0.435-1.53)	0.526		
Gender				
Male	Reference	-		
Femal	0.964(0.535-1.736)	0.903		
TNM staging				
I/II	Reference	-	Reference	-
III/IV	4.359(2.107-15.27)	*0.005*	3.537(0.897-11.67)	*0.013*
Pathological grade				
I	Reference	-		
II	1.815(0.649-5.076)	0.256		
III	-	-		
Lymph node metastasis				
No	Reference		Reference	-
Yes	1.987(1.112-3.551)	*0.02*	1.385(0.798-2.297)	0.067
Tumor size				
<=5cm	Reference			
>5cm	0.993(0.552-1.786)	*0.981*		
T stage				
T1/T2	Reference	-	Reference	-
T3/T4	4.277(1.526-11.985)	*0.006*	3.038(0.924-9.451)	*0.031*
Distant metastasis				
No	Reference	-	Reference	-
Yes	16.247(7.55-36.38)	0.000	6.883(2.318-20.43)	*0.001*
CEA				
Normal	Reference	-	Reference	-
High	3.27(1.572-6.801)	*0.002*	1.934(0.840-4.344)	*0.121*
CA199				
Normal	Reference	-		
High	0.522(0.233-1.171)	0.115		
FOXP2				
Low	Reference	-	Reference	
High	0.69(0.442-0.892)	*0.02*	0.757(0.499-1.041)	*0.037*
Caspase-1				
Low	Reference	-	Reference	-
High	0.791(0.506-0.901)	0.042	1.012(0.687-1.515)	0.381

Italic values indicate statistical significance when *P<0.05*
