# Supplementary material for: Low expression of FOXP2 predicts poor survival and targets caspase-1 to inhibit cell pyroptosis in colorectal cancer
Source: J Cancer. 2022 Jan 16;13(4):1181–92. doi: 10.7150/jca.62433 (PMC8899368; doi:10.7150/jca.62433)
Supplement: Supplementary file 1 — Supplementary figure and information. [file jcav13p1181s1.pdf]

Homo sapiens chromosome 11, GRCh38.p13 Primary Assembly

NCBI Reference Sequence: NC_000011.10

GenBank Graphics

>NC_000011.10:c105025442-105024442 Homo sapiens chromosome 11, GRCh38.p13 Primary Assembly

AGAGTGTTTGACTTAATCTGTCAGACCCAAACTGGCTATTGGCACCTTTCTTTCCCAGA
ACTTAAAGTAAAATAAAATATAAAAATTTAAAAAAGAAAATCAGACTTACAAACATAGC
AATGTTATTAATTACTTTCTTGAACAAAAATTTATTTTGTGCCATTTTTAAAAAAAGAAA
AATCGTTTGCTGGGCTTTCTGCTTTGGGGATGTGATATAAGTTACAGCAGGTATCTGACT
TCAAATAATTTAGAGCTCAGCAGGAGAGACGGGGGAAGACAGCTAGTTATGACAGCG
ACAGTGTAGTGTGATAAAGAACTTTTCCCAAATCCCACAGTTTGCTACTGACAAATCCA
TGAACTTGCATGTTTCTACCCTTTGCAAAAGTATTTAAGACAAAGTAAGTTTTGAATCA
ACCCTCAGTAGTTCACAGACTTCTTGAAAAGATAAGACAGCCATCCAAAATGAAGCGC
TTGGCAGATGGTGCCTGACACCCTGAAGAAACTCTAGGATGCATGGTGTGCACAGTGA
AGTCCTGGCATGGTGCAGAGAAAACGAGTTTGGGGTGAAATCATGCACTCAGGGATGT
TAAGATTTAGGGAGGATTCGAAGAACTACCCCTTACTTGCAAATATGTTTTTTTCTAATA
TTTAACAGATTAAATGAGAGTTAATTTCTGACTTCTTAAACACCTTCATAGTTGAACACA
TTACAGTCTCTCTCTCTCTCTCTTTTTTTTTTTTTTACCTTTTATTAACTGTTTCTCTGGG
CATATCTCTTCTGTGTTATCATGCATACTCTACTACAACCTGAGACTACATCTGTGATCA
GTCTTTGCTCAGAGAATATCAAAAAGGAGAAGTGAATTGCCACCCTAAGTAAAAATTA
ACTGTTAAAGCAGTTTTAGTCAGTTGAAGAACAGCATTCCAGGAAGTAGATAAGAGGA
TTCCTTCAGAACTGTCTCCAATCAAAGCGTAAGATGACAGGGAGATGGGGAGGAGA

Supplementary Figure 1: A-C, A heat plot was generated to show the differentially expressed genes by analysing GSE44904 and GSE44988.

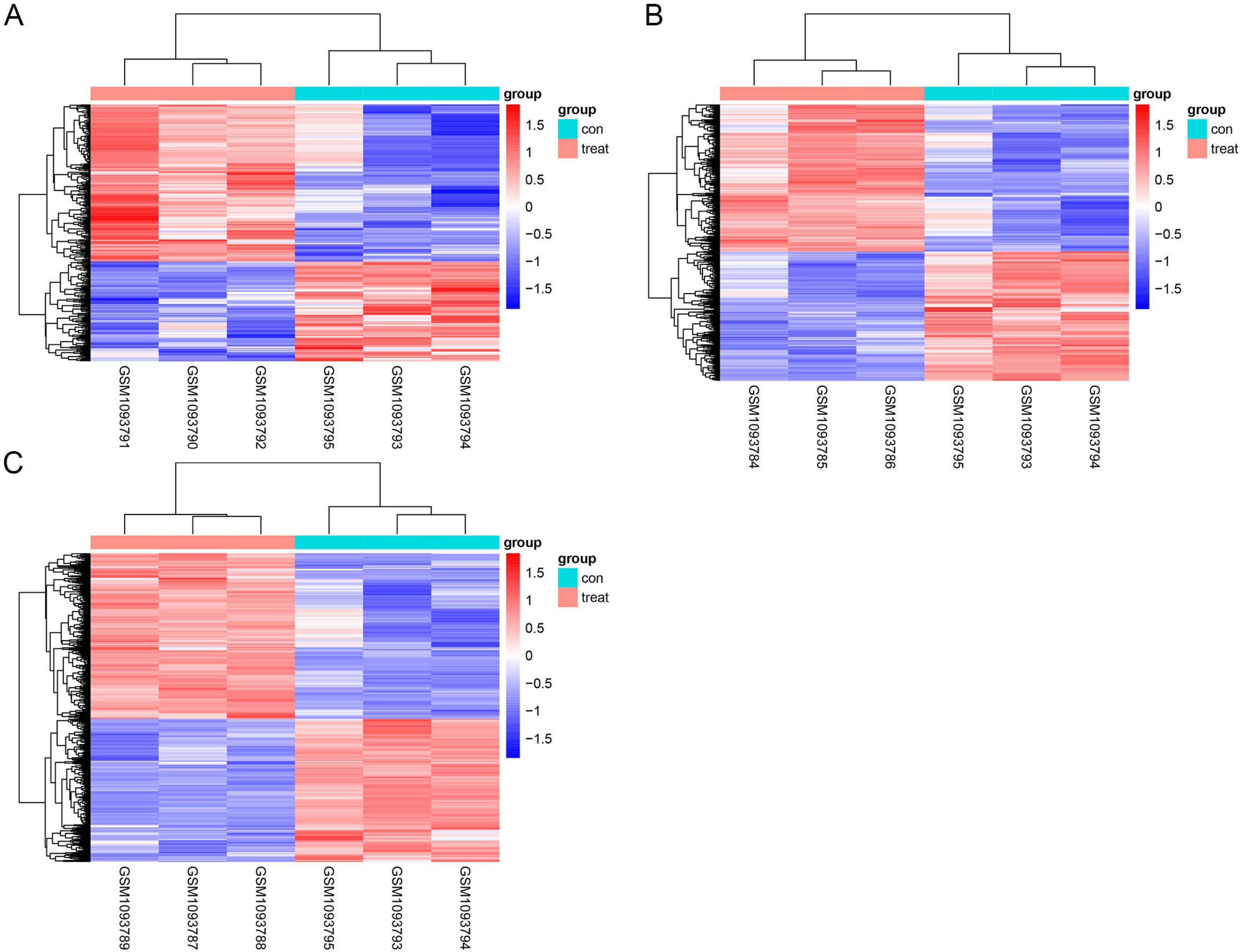
A
group
group
con
treat
1.5
1
0.5
0
−0.5
−1
−1.5
GSM1093791
GSM1093790
GSM1093792
GSM1093795
GSM1093793
GSM1093794
B
group
group
con
treat
1.5
1
0.5
0
−0.5
−1
−1.5
GSM1093784
GSM1093785
GSM1093786
GSM1093795
GSM1093793
GSM1093794
C
group
group
con
treat
1.5
1
0.5
0
−0.5
−1
−1.5
GSM1093789
GSM1093787
GSM1093788
GSM1093795
GSM1093793
GSM1093794
